# Utilization of the high spatial-frequency component in adaptive beam shaping by using a virtual diagonal phase grating

**DOI:** 10.1038/s41598-019-40829-7

**Published:** 2019-03-15

**Authors:** Yoshiki Nakata, Kazuhito Osawa, Noriaki Miyanaga

**Affiliations:** 1 Institute of Laser Engineering, Osaka University, 2-6 Yamadaoka, Suita, Osaka 565-0871 Japan; 2 Institute for Laser Technology, 2-6 Yamadaoka, Suita, Osaka 565-0871 Japan

## Abstract

A square flattop beam is a fundamental shape that is in high demand in various applications, such as ultra-high-power lasers, uniform surface processing and medical engineering. In this experiment, a new and simple scheme of the adaptive beam shaping system to generate a square flattop shape with high uniformity and edge steepness using virtual diagonal phase grating encoded on a spatial-light modulator and a 4*f* system is proposed. The grating vector *k*_*g*_ is non-parallel to the normal vectors *k*_*x*_ and *k*_*y*_ of the objective beam profile to be extracted; thus, the residual and extracted components hit separately on the Fourier plane of the 4*f* system. Consequently, using a spatial-frequency filter passing components parallel to *k*_*x*_ and *k*_*y*_, the residual components are blocked by the filter without loss of the high spatial-frequency domain of the extracted component. When the width of the filter was 1.0 mm, the edge of the shaped beam increased in height within 20 μm, which is less than 20% of that obtained with conventional vertical phase grating.

## Introduction

Beam shape is key to realising a laser’s potential abilities, and various beam shaping techniques have been developed for different applications. A square flattop beam is a fundamental shape that is in high demand in ultra-high-power laser facilities that conduct experiments into inertial fusion as a new energy source^1–4^ and laboratory astrophysics^5^. In such facilities, a large square flattop beam is amplified via glass^6^ and/or an optical parametric chirped pulse amplifier^7–10^. Here, the shape and flatness determines the maximum power of the ultra-high-power laser system where the fill factor to the amplifier and damage to the optics at a peak region govern the limits^10^. In addition, the performance of area laser processing, such as scribing^11^, repairing^12^, interference processing^13–15^ and skin therapy^16^, is improved using a square flattop beam.

**Figure 1:**
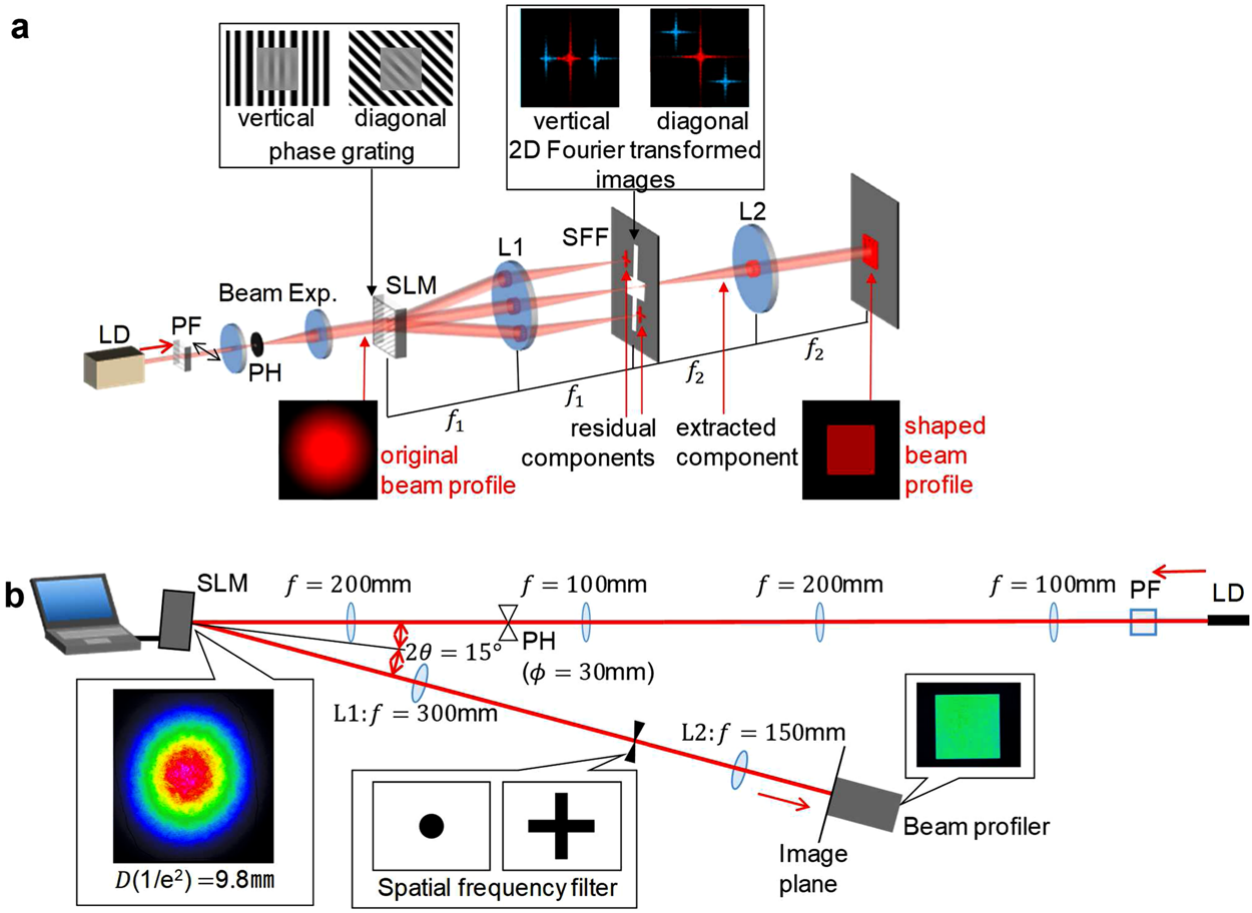
Experimental layout. (**a**) General scheme. (**b**) Actual Implementation. Gaussian beam with a beam diameter of *D* = 9.8 mm (at 1/*e*^2^). A vertical or diagonal phase grating is overlapped via a reflective SLM and imaged on a screen or a beam profiler at a magnification factor of *M* = *f*_2_/*f*_1_.

**Figure 2:**
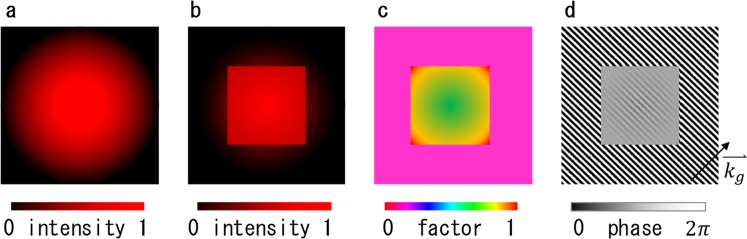
Scheme for shaping beams to a flattop square via phase gratings. (**a**) Original beam profile. (**b**) Target beam profile. (**c**) Transfer function. (**d**) Phase grating reflecting the transfer function and the output curve as a function of Δ*ϕ*.

Static and adaptive beam shaping methods have been developed for various applications. Refractive beam shapers^17,18^, microlens arrays^19,20^ and computer-generated holograms (CGH)^21^ that distribute the original laser energy to a targeted field are conventional static beam shaping methods. However, with such methods, edge steepness and flatness are not high. In addition, such methods do not consider the optical length inside the beam and/or divided beams; therefore, the pulse shape and wavefront become deformed. Various adaptive filtering techniques have also been developed. For example, CGH on a phase-only spatial-light modulator (SLM) imaged using a Fourier transforming lens can generate arbitrary patterns on a far-field^22^. However, this method also has difficulty with the pulse shape and wavefront due to the distribution of the optical length. In addition, a system containing two-phase-only SLMs is designed, in which the first redistributes the light and the second re-collimates the beam. The wavefront was improved, but it has difficulties in flatness and complexity of the system^23^. Moreover, an adaptive beam shaping technique that uses phase grating encoded on an SLM with spatial-frequency filtering in the Fourier plane in a 4*f* system has been developed^6,9,24–28^, as shown in Fig. 1. With this technique, phase grating diffracts the residual components to extract the component regenerated on the image plane with a square flattop beam profile. Here, the scheme of this technique is explained in Fig. 2. The original beam profile (Fig. 2a) is compared to the desired beam profile (Fig. 2b) to generate a transfer function image (Fig. 2c) and to map the ratio of the extracted beam intensity to the original beam intensity. The transfer function image is converted to a phase grating image (Fig. 2d), as follows. Local ratio of the image is converted to phase depth Δ*ϕ* by using the intensity curve of the extracted beam as a function of the phase depth Δ*ϕ*, which is shown in Supplementary Fig. S6 (see Supplementary Information, Sect. 3). As a result, a phase grating, in which the diffraction efficiency is controlled spatially, to form the desired beam profile is created.

**Figure 3:**
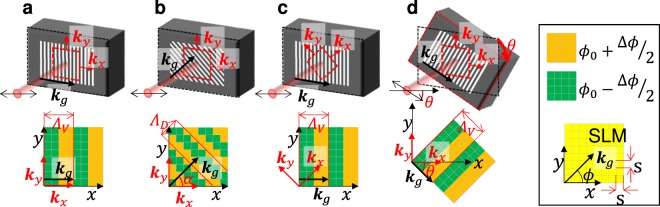
Phase grating and square flattop beam profile to be extracted on SLM. *k*_*g*_ is the grating vector, and *k*_*x*_ and *k*_*y*_ are the normal vectors of the desired beam profile. (**a**) Vertical phase grating on the SLM with desired square beam profile. (**b**) Diagonal zigzag phase grating with desired square beam profile. (**c**) Vertical phase grating with desired diamond beam profile. (**d**) Vertical phase grating on a slanted SLM with desired square beam profile. The upper panels illustrate the SLM settings, the phase grating on the active area of the SLM, the desired beam shape (dotted red square) and the incident beam. The double-headed arrows indicate the polarisation adjusted to the direction specified for the SLM. The lower panels illustrate the phase gratings on the corresponding SLMs. The right inset explains the pixel structure on the *xy*-plane, and the colours indicate the phase on the pixels. *Λ*_*D*_, *Λ*_*V*_: phase grating period, *s*: pixel size, *α*: phase grating angle in the x*y*-plane and *θ*: SLM angle from the horizontal setting.

**Figure 4:**
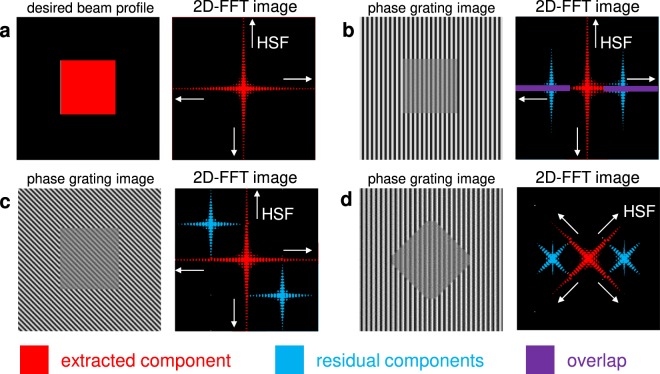
Desired square flattop beam profile, phase grating images and corresponding 2D-FFT images. (**a**) Desired square flattop beam profile on the image plane, which is extracted from the phase grating on the SLM and its 2D-FFT image. (**b**) Vertical phase grating image to regenerate the square beam profile and its 2D-FFT image. (**c**) Diagonal phase grating image to regenerate the square beam profile and its 2D-FFT image. (**d**) Vertical phase grating image to regenerate the diamond beam profile and its 2D-FFT image. The phase grating images are schematic and projected as they appear on the SLM, and the 2D-FFT images are projected as they appear in the Fourier plane from the SLM side. Areas of the extracted component, residual components and their overlap are shown in red, blue and violet, respectively.

In the conventional scheme, vertical or horizontal phase grating is encoded according to the SLM pixel matrix, as shown in Fig. 3a. In this case, grating vector *k*_*g*_ is parallel to the normal vectors *k*_*x*_ or *k*_*y*_ of the desired beam profile. The extracted and residual components are transformed to the Fourier plane via a convex lens L1, as shown in Fig. 1, and the residual components are blocked. Note that the spatial-frequency distribution of the desired beam profile in the 2D-FFT image, i.e. the extracted component, widens horizontally and vertically along the normal vectors *k*_*x*_ or *k*_*y*_, as shown in Fig. 4a (see Supplementary information, Sect. 1)^29^. In addition, the residual components are distributed horizontally and vertically in the 2D-FFT image, as shown in Fig. 4b. Therefore, the extracted and residual components overlap in the Fourier plane, as indicated by the violet areas in the figure. As a result, the high spatial-frequency (HSF) component of the extracted component is blocked with the residual components, and the steepness of the resultant beam shape is limited relative to the phase grating resolution. Note that this limitation is inherent in conventional adaptive beam shaping systems.

In this paper, we adopted virtual diagonal phase grating encoded on an SLM to utilize the HSF component of a square beam fully, and overcame the limitation in the edge steepness and resolution in the conventional adaptive beam shaping method.

## Principle

Here, the problem is the overlapping spatial-frequency distributions of the extracted and residual components in the Fourier plane, as shown in Fig. 4b. To address this issue, there are three possible configurations to avoid overlapping extracted and residual components^27^. In the first configuration, the phase oscillates in the diagonal direction to form a virtual diagonal “zigzag” phase grating with α = 45°, as shown in Fig. 3b. As a result, the direction of ***k***_***g***_ differs from the normal vectors *k*_*x*_ and *k*_*y*_ of the desired beam profile. The residual components are diffracted in the orthogonal plane perpendicular to the virtual diagonal zigzag grooves of the phase grating, and the residual and extracted components are separated in the Fourier plane, as shown in Fig. 4c. In this scheme, the extracted HSF component can be transmitted through the spatial-frequency filter (SFF); however, the residual components are blocked.

In the second configuration, the beam has a diamond shape in the active area of the SLM, as shown in Fig. 3c; therefore, the directions of *k*_*x*_ and *k*_*y*_ are diagonal and differ from *k*_*g*_, which also results in separation of the extracted and residual components in the Fourier plane, as shown in Fig. 4d. However, a diamond shape is not typically used in laser systems^6–9^. In the third configuration (Fig. 3d), a slanted setting of the SLM results in *k*_*g*_ differing from *k*_*x*_ and *k*_*y*_. Note that this setting is equivalent to that shown in Fig. 3c; however, the resultant beam shape is square. Here, the polarisation must be rotated according to the slanting angle *θ*, which results in complexity and poor adaptability to other instruments. From a practical perspective, the diagonal grating shown in Fig. 3b is selected in this paper and compared to the vertical grating shown in Fig. 3a.

### Experimental scheme

The experimental setup is shown in Fig. 1 (see Supplementary Section 2 and Methods). Here, a continuous wave laser beam from a laser diode (LD) at 780 nm with a Gaussian beam profile was used. The vertical and diagonal phase grating was encoded to the beam using a reflective SLM. The beam was transformed by a 4*f* system comprising two convex lenses with spatial filtering in the Fourier plane. The shaped beam profile was then observed in the image plane.

## Results and Discussion

**Figure 5:**
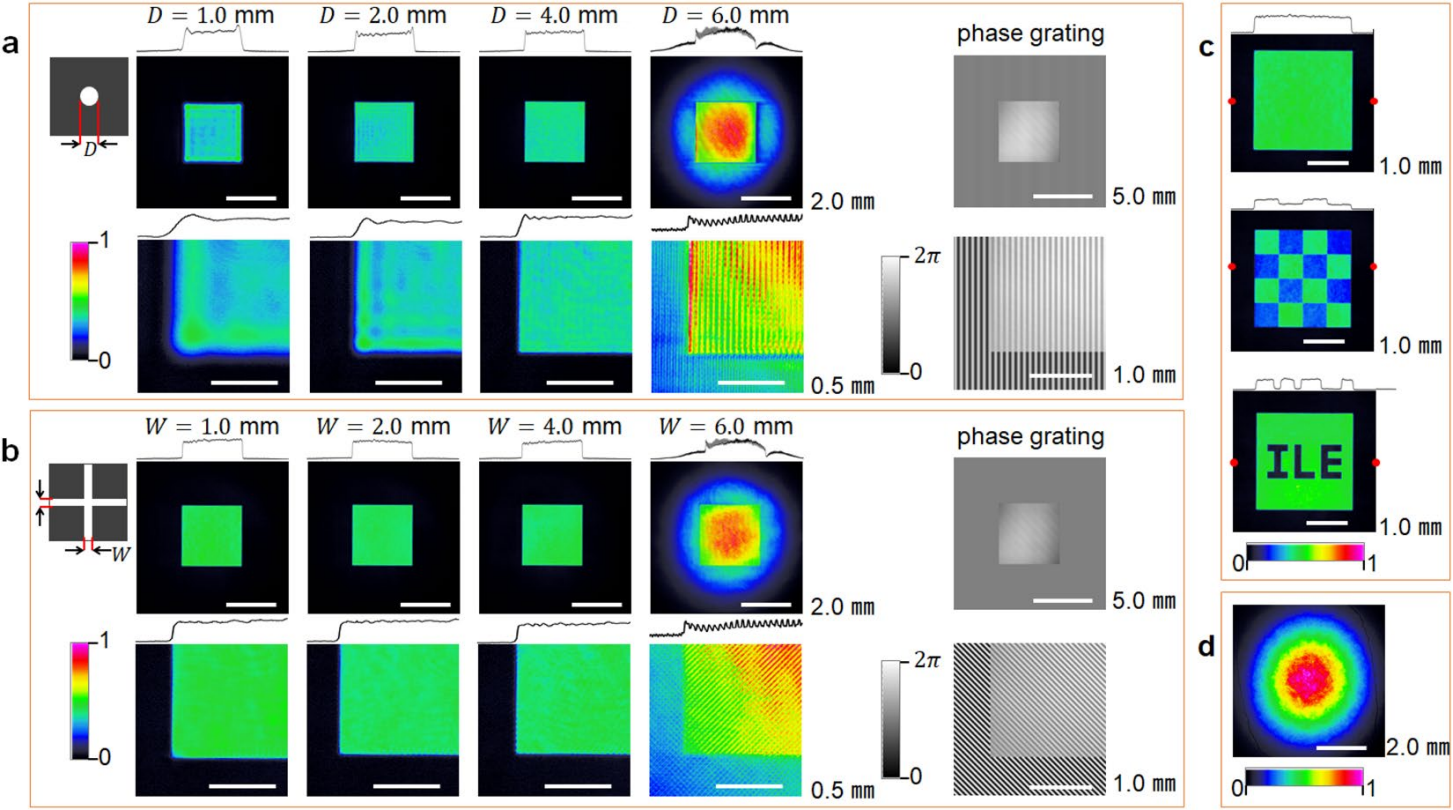
Flattop and square beam profiles as a function of the shape and size of the SFFs, and corresponding magnified views of the lower left corners. (**a**) Vertical phase grating. (**b**) Diagonal phase grating. The upper insets show the horizontal cross-sectional graphs across the centre of the beams, and right insets show the corresponding images of phase gratings. The gray scales show the phase. (**c**) Addition of checker and ‘ILE’ pattern with diagonal phase grating, and cross-sectional graphs across red points. (**d**) Original beam profile. The colour bars show signal intensity.

An experiment was conducted to demonstrate the concept. Figure 5a shows a beam profile with vertical phase grating as a function of the diameter of the SFF. Here, the SFF was placed in the Fourier plane and the centre was co-aligned to the focal point of the zeroth-order beam. The first-order diffracted beam was 2.6 mm from the centre. Note that the graph above each image is the corresponding horizontal cross-sectional graph across the centre of the beam. With an SFF diameter of *D* = 1.0 mm, a wavy structure was observed due to the cut of the HSF component of the square flattop beam profile. It was observed that quality improved as the diameter increased to D = 2.0 and 4.0 mm, which is clearly shown in the magnified views of the lower left corners. For D = 6.0 mm, first-order diffracted beams were transmitted through the SFF; therefore, the region outside the square appears and the grating structure can be observed.

Figure 5b shows the beam profile with diagonal phase grating as a function of width *W* of the cross-shaped SFF. The shape was designed to transmit the HSF of the extracted component and block the residual components, as shown in Fig. 4c. The centre of the SFF was co-aligned with the focal point of the zero-order beam. In addition, the first-order diffracted beams were positioned to lie in the second and fourth quadrants of the Fourier plane, as shown in Fig. 4c. The first-order diffracted beam was located 4.2 mm from the centre of the SFF. When the width of the SFF was *W* = 1.0 mm, quality improved significantly compared to the vertical phase grating case with *D* = 1.0 mm, which is shown in Fig. 5a, because the HSF can be transmitted through the cross-shaped SFF. Note that beam quality was also good with *W* = 2.0 and 4.0 mm. At *W* = 6.0 mm, first-order diffracted beams were transmitted through the SFF, which made the appearance of the diagonal comb structure and beam shaping incomplete.

Figure 5c shows the beam profiles with the checker and ‘ILE’ patterns with diagonal phase grating at *W* = 2.0 mm. These images clearly demonstrate that the scheme is useful for patterning on a flattop beam at high resolution. The point is that, in these designs, normal vectors of any sides are not parallel to grating vector *k*_*g*_.

**Figure 6:**
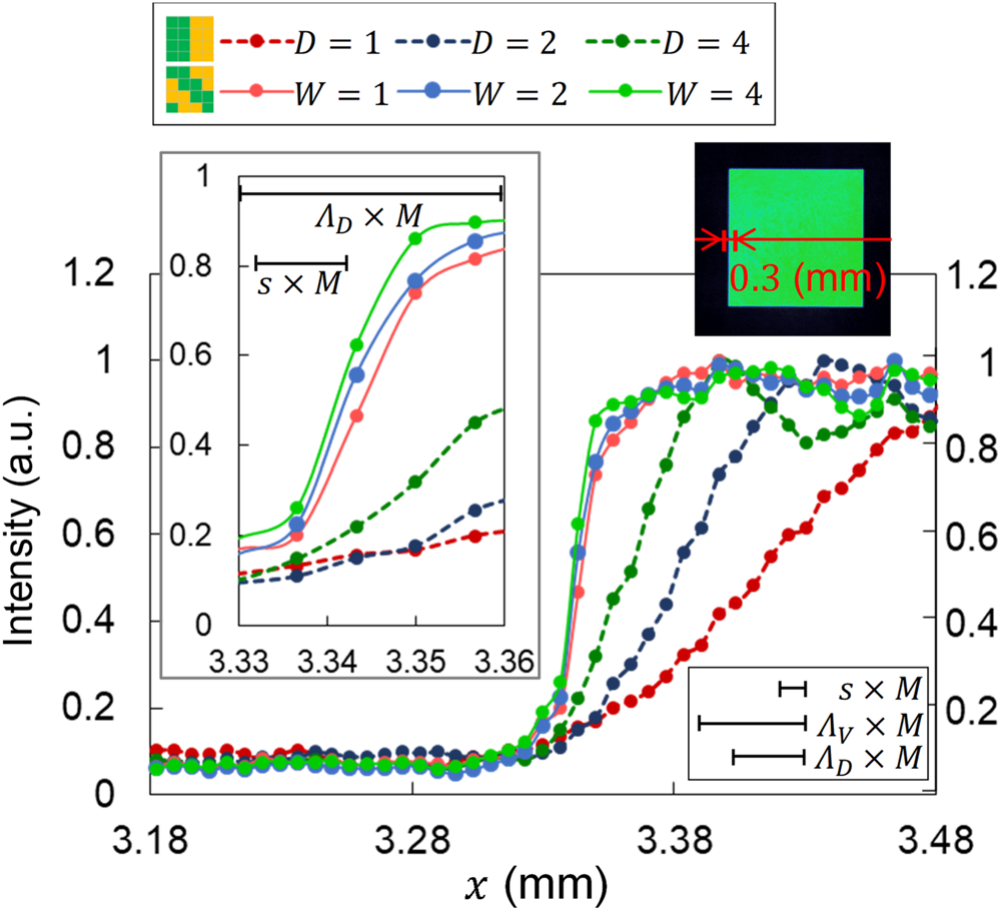
Cross-sectional graphs in 0.3 mm of the left edge of the shaped beam profiles. The two patterns in the legend indicate vertical and diagonal phase gratings. The upper right inset shows the area of the cross section. The upper left inset shows the *x* value (3.33 to 3.36 mm). Horizontal lines with caps show the size of the pixel of SLM (*s*) and the periods of vertical and diagonal gratings (*Λ*_*V*_ and *Λ*_*D*_) magnified by a factor of *M* = 0.5 considering the magnification factor of the 4*f* system.

The advantage of the proposed scheme is the steepness and resolution of the edge of the regenerated beam profile. Figure 6 shows cross-sectional graphs of the left edge of the shaped beam profiles shown in Fig. 5. The graphs are along the beam centre. Here, the graphs of edge steepness are plotted for *D*, *W* = 1.0, 2.0 and $4.0$ mm because the beam profiles with *D*, *W* = 6.0 mm were destroyed due to the inclusion of the residual components. Note that the points in the graphs reflect the pixels in the beam profiler.

**Figure 7:**
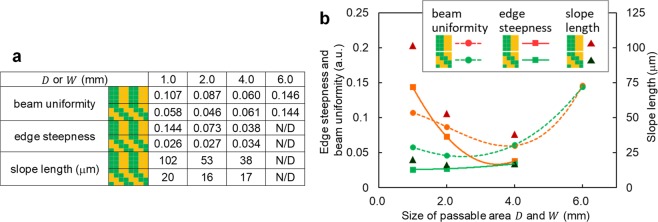
Beam uniformity, edge steepness and slope length as a function of the sizes of the SFFs. (**a**) Table and (**b**) graph.

It appears that the steepness is quite different for the vertical and diagonal phase gratings. In the vertical phase grating case, the steepness is better with larger *D*; however, the slope rises slowly even with the best case of *D* = 4.0 mm. The slope length of projection to the *x* axis with intensity from 20% to 80% is summarised in Fig. 7. As can be seen, the slope is 38 μm with *D* = 4.0 mm and is nearly the same as that of the grating period on the beam profiler (*Λ*_*V*_ × *M* = 40 μm), which is indicated by the horizontal line with caps in Fig. 6. Under this condition, the vertical grating structure appears faint on the beam profile, as shown in the magnified view in Fig. 5a. This is due to the inclusion of a part of the residual components through the SFF.

In the diagonal phase grating case, steepness is higher compared to the vertical phase grating case. Here, the slope length is less than 20 μm for each width of the SFF. As is clearly shown in the upper left inset in Fig. 6, the length is shorter than the period of the phase grating on the beam profiler (*Λ*_*D*_ × *M* = 28.3 μm). This reflects utilization of the HSF component beyond the phase grating frequency, which is enabled by the proposed scheme for the first time.

Beam uniformity and edge steepness as a function of the sizes of the SFFs are summarised in Fig. 7 using a calculation that is compatible with the ISO 13694:2000(E) Standard (see Supplementary Information, Sect. 4). Here, beam uniformity ranged from zero to one, with a perfect flat top beam having a value of zero, and the edge steepness ranged from zero to one, with vertical edges having a value of zero. In the vertical phase grating case, the uniformity and edge steepness were better at greater *D* values and best at *D* = 4.0 mm. Therefore, quality improved as the HSF component was transmitted through the SFF. At *D* = 6.0 mm, the uniformity was reduced due to the vertical comb structure. In the diagonal phase grating case with *W* = 1.0 mm, beam uniformity improved to 54% of that with vertical phase grating with *D* = 1.0 mm, and the edge steepness improved to 18% of that with vertical phase grating. Note that these values were similar or better at *W* = 2.0 mm. It appears that these values decrease somewhat at *W* = 4.0  mm, which is likely due to the inclusion of part of the first-order diffracted beam.

The results indicate that diagonal phase grating with *W* = 2.0 mm works best to obtain best beam uniformity, edge steepness and short slope length simultaneously. In addition, the sweet spot is wide at *W* = 1.0–2.0 mm. This allows quick alignment of the system and results in a stable system. Note that such high quality beam shaping can be achieved without iterative feedback, even though the vertical phase grating method achieved lower quality using iterative feedback^24^.

In this scheme, residual components are blocked on a Fourier plane to shape a beam. The system in this experiment was not optimized to the efficiency, and it was 23%. In general, the beam shaping system is introduced at front-end subsystem in multiple amplification systems in the case of ultra-high-power laser facilities^9^.

## Conclusion

We have demonstrated a beam shaping method that uses virtual diagonal zigzag phase grating encoded on an SLM and 4*f* system. This original and simple scheme utilises the HSF component of a square beam. The results obtained with the proposed scheme demonstrate short slope length, high uniformity and edge steepness simultaneously. In addition, the wider sweet spot of the SFF size results in quick and easy settings without iterative feedback; therefore, the proposed scheme affords good system stability. Note that the proposed scheme is not restricted to the use of Gaussian beam profiles, i.e. it can be adapted to any laser system and a wide range of applications, where spatial distribution is critical. Note that the SLM is the only cost-consuming component that does not have to be specialised relative to resolution. In addition, conventional adaptive beam shaping systems can be improved significantly without additional costs. We consider that these advantages will result in new scientific discoveries in a wide range of applications that use beam shaping by improving both resolution and accuracy.

## Methods

### 2D-FFT

The 2D-FFT method used in the simulation of the images in Fig. 3 is explained in Supplementary Information, Sect. 1 ‘2D-FFT method’.

### Experimental setup

The experimental setup is shown in Fig. 1 (see Supplementary Information, Sect. 2). A 780-nm beam from a focused LD module (85-231, Edmund) passed through a polarisation filter (PF) with a hole diameter of 30 μm (84-065, Edmund) to form a Gaussian beam. The magnification factor was *M* = (200/100)^2^ = 4.0, which was selected such that the beam filled the active area of the SLM. The vertical and diagonal phase grating overlapped the beam via an SLM (LCOS-SLM X-10468-02, Hamamatsu Photonics K. K.) with an incident angle of *θ* = 7.5°. The phase grating was encoded via a DVI connection with a PC. The specifications of the SLM are summarised in Supplementary Table S1 (see Supplementary Information, Sect. 2). The beam shape was imaged on the beam profiler (LaserCam-HR, Coherent). The specifications of the beam profiler are summarised in Supplementary Table S2 (see Supplementary Information, Sect. 2). The demagnification factor between the SLM and beam profiler was $M=\frac{150}{300}=0.5$ in consideration of the size of the imaging element. The diameters of the lenses were *ϕ*_*f*=300_ = 50 mm and *ϕ*_*f*=150_ = 25.4 mm. Note that all lenses were achromatic. The filter was made from white cardboard with a printed grid for precise cutting.

### Expression of amplitude control by depth of phase grating

The theoretical formula of the beam intensity of the extracted beam as a function of the phase depth of the grating and its experimental derivation is explained in Supplementary Information, Sect. 3. Phase grating with a uniform phase depth Δ*ϕ* (Figs S3 and S4) was encoded to the Gaussian beam, and extracted beam profiles were imaged, as shown in Fig. S5. The period of the vertical phase gratings was *Λ* = 80 μm (4 pixels) and the period of the diagonal phase gratings was *Λ* = 56.6 μm ($2\sqrt{2}$ pixels). The intensity of the extracted beam averaged over a circle at the beam centre with diameter of 1 mm is plotted for vertical and diagonal gratings in Fig. S6. Note that the graphs were normalised to their maximum value at Δ*ϕ* = 0. They are attenuated to 3.2% and 3.8% with the vertical and diagonal gratings, respectively. The fitted curves were modelled as follows.

1 $$\begin{array}{c}{I}_{vertical}({\Delta }\varphi )=1.47+98.87\,\cos \,{(3.16-0.38{\Delta }\varphi )}^{2}\end{array}$$

2 $$\begin{array}{c}{I}_{diagonal}({\Delta }\varphi )=3.16+97.11\,\cos \,{(3.13+0.31{\Delta }\varphi )}^{2}\end{array}$$

The transfer function image, as shown in Fig. 2c to map the ratio of the extracted beam intensity to the original beam intensity, was converted into a phase grating image using these curves, in which the depth of phase grating Δ*ϕ* was distributed according to Eqs (1) and (2).

## Supplementary information

Supplementary information
