# Supplementary material for: Utilization of the high spatial-frequency component in adaptive beam shaping by using a virtual diagonal phase grating
Source: Sci Rep. 2019 Mar 15;9:4640. doi: 10.1038/s41598-019-40829-7 (PMC6420635; doi:10.1038/s41598-019-40829-7)
Supplement: Supplementary file 1 — Supplementary information [file 41598_2019_40829_MOESM1_ESM.pdf]

Supplementary information for

Utilization of the high spatial-frequency component in adaptive beam shaping by using a virtual diagonal phase grating

Yoshiki Nakata[1*], Kazuhito Osawa[1], Noriaki Miyanaga[2,1]

[1] Institute of Laser Engineering, Osaka University, 2-6 Yamadaoka, Suita, Osaka 565-0871 Japan.

[2] Institute for Laser Technology, 2-6 Yamadaoka, Suita, Osaka 565-0871 Japan

Correspondence to; nakata-y@ile.osaka-u.ac.jp

This file includes;

Section 1. 2D-FFT method

Section 2. Experimental scheme

Section 3. Expression of amplitude control by the depth of phase grating and experimental derivation

Section 4. Numerical evaluation method for flatness and edge steepness

1. 2D-FFT method

The 2D-FFT images depict the overlapping of phase gratings on a Gaussian beam. The electric field strength and phase distribution are superimposed at the centre of a black base image with 1024×1024 pixels, which is expressed as follows;

$$E_0(i,j) = \sqrt{intensity(i,j)} \qquad (1)$$

$$E(i,j) = E_0(i,j) \times exp\big(k \times grating(i,j)\big) \qquad (2)$$

$$(i,j = 1,2,3,\cdots 1024),$$

where $intensity(i,j)$ expresses the intensity on the corresponding pixel, $grating(i,j)$ expresses the phase, and k is the imaginary unit. The period for the vertical gratings is $\Lambda = 4$ pixels and the corresponding period for the diagonal grating is $\Lambda = 2\sqrt{2} = 2.83$ pixels as shown in Fig. 3a and 3b. The matrix is transformed by Mathematica © Wolfram. The result is transferred to an image by squaring $E(i,j)$ pixel to pixel.

2. Experimental setup

The experimental setup is shown in Figure 1. The specification of the beam profiler (LaserCam-HR, Coherent) is summarized in Table S2. The specification of the SLM (LCOS-SLM X-10468-02, Hamamatsu Photonics K. K.) is summarized in Table S1.

Pixel pitch	20 μm
Input signal	Digital Video Interface (DVI-D)
DVI signal format	SVGA (800 x 600 pixels)
DVI frame rate	60 Hz
Number of input levels	256 (8 bits) levels
Effective area	15.8 × 12 mm
Spatial resolution max.	25 lp/mm (20μm/pixels)

Fill factor	98 %
Readout light wavelength	800 ± 50 nm
Light utilization efficiency	97 %
Measurement condition	λ=785 nm

Table S1. Specification of the SLM (LCOS-SLM X-10468-02, Hamamatsu Photonics K. K.)

Sensor	2/3", 1.3 Megapixel, CMOS
Wavelength Range	300-1100 nm
Matrix size	1280 x 1024
Pixel pitch	6.7 x 6.7 μm
Active Area	8.6 x 6.9 mm
Signal to noise	1000:1
Digitization Depth	10 Bits
Frame Rate	27 FPS - Live Mode, 10 FPS With Calculations

Table S2. Specification of the beam profiler (LaserCam-HR, Coherent)

3. Expression of amplitude control by depth of phase grating and experimental derivation

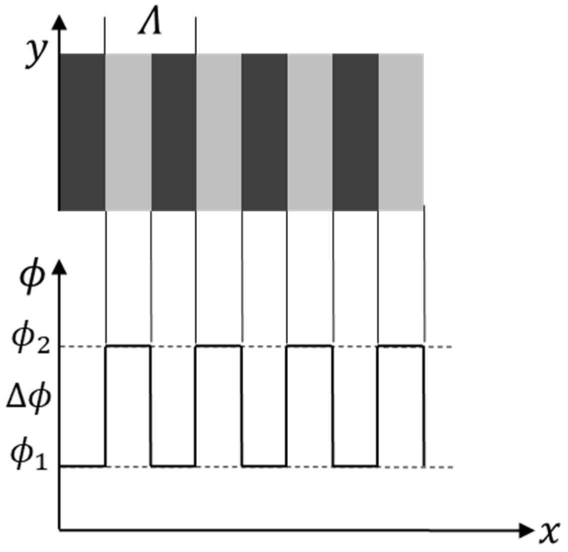

Figure S1. Schematic of phase grating with uniform phase depth $\Delta\phi$. x, y are the

coordinates in the active area of the SLM.

Considering the uniform vertical phase grating as shown in Fig. S1. ϕ_1, ϕ_2 are the phase encoded by a SLM, $\Delta\phi = \phi_2 - \phi_1$, Λ is the period of the phase grating, and x and y are the coordinates on the SLM. The comb phase grating as shown in the figure is expressed in the following Equations (3), (4) and (5);

$$E' = E_1 + E_2 \qquad (3)$$

$$E_1 = E_0 \sum_n \delta(x - n\Lambda) \otimes rect_{\frac{\Lambda}{2}}(x) \exp(j\phi_1) \qquad (4)$$

$$E_2 = E_0 \sum_n \delta(x - n\Lambda) \otimes rect_{\frac{\Lambda}{2}}\left(x - \frac{\Lambda}{2}\right) \exp(j\phi_2) \qquad (5).$$

Here, n is the spatial frequency and E_0 is the complex amplitude. E' oscillates along the x-direction, δ is the Dirac function, and $\otimes$ expresses the convolution. For example, E' is plotted as shown in Fig. S2.

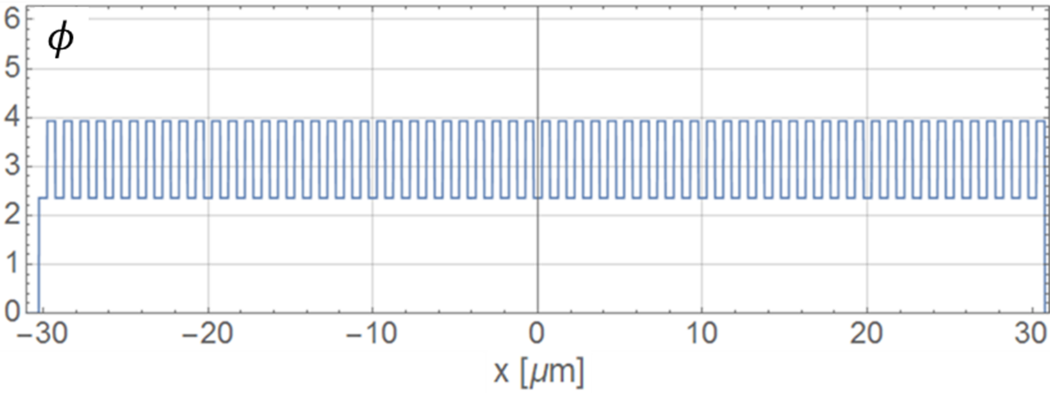

Figure S2. Example plot of electric field of an oscillating phase grating. E_0=1V/m、Λ=1μm, $-30 \leq n \leq 30$, $\Delta\phi = \frac{\pi}{2}$.

Assuming a cut of the residual component at the Fourier plane, the field intensity of the extracted beam is expressed as follows[1];

$$\tilde{E}(0) \propto E_0 \cos\left(\frac{\Delta\phi}{2}\right) \times \exp\left(j\frac{\phi_1 + \phi_2}{2}\right) \qquad (6)$$

The expression (4) shows that the field intensity is controlled by the cosine function of $\Delta\phi$. The formula assumes a perfectly rectangular phase grating shape, and infinite size of the phase grating in the expression is obviously finite in the experiment. In addition, the SLM has the deviation of phase shift at the slits between the pixels. Thus, the real attenuation curve deviates from the theory and should be measured experimentally.

The extract beam intensity as a function of phase depth $\Delta\phi$ was measured as follows. The uniform vertical and diagonal phase grating, which is shown in Fig. S3 and S4, is encoded over the whole area of the SLM. The period of the vertical phase gratings was $\Lambda = 80$ μm (4 pixels) and the period of the diagonal phase gratings was $\Lambda =$ 56.6μm ($2\sqrt{2}$ pixels). The first-order diffracted beam appeared 2.6 mm and 4.2 mm from the 0th-order beam, respectively. Here, the attenuation factor in the case of the vertical phase grating with $\Lambda = 40$μm (2 pixels) is 44%[2], which is not enough for filtering the residual component. Therefore, the $\Lambda = 80$ μm was adopted, which results in enough attenuation as explained below. The full view size is 800×600 pixels to adjust the pixel format of the SLM.

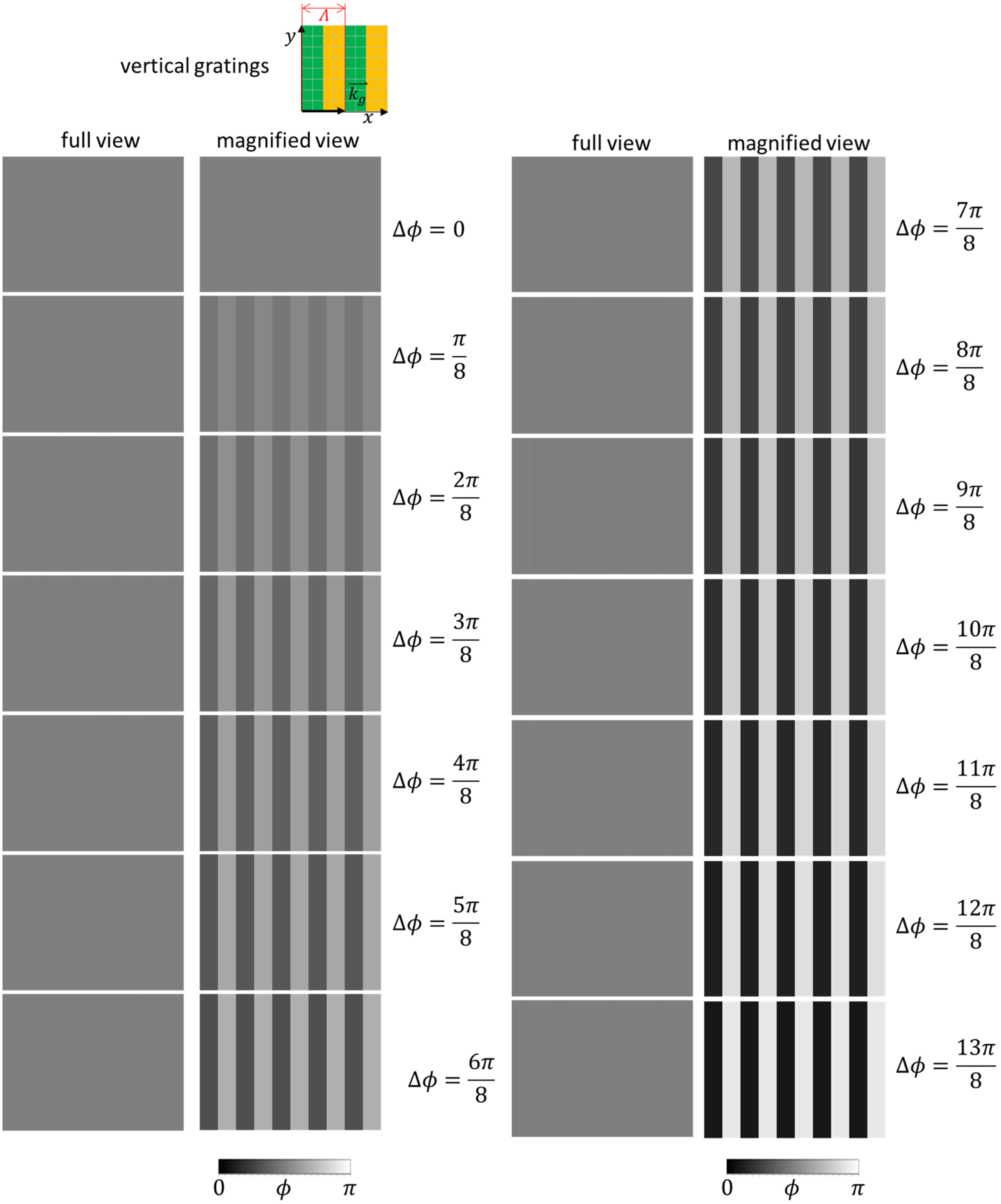

Figure S3. Vertical phase gratings for the measurement of extract beam intensity as a function of phase depth $\Delta\phi$.

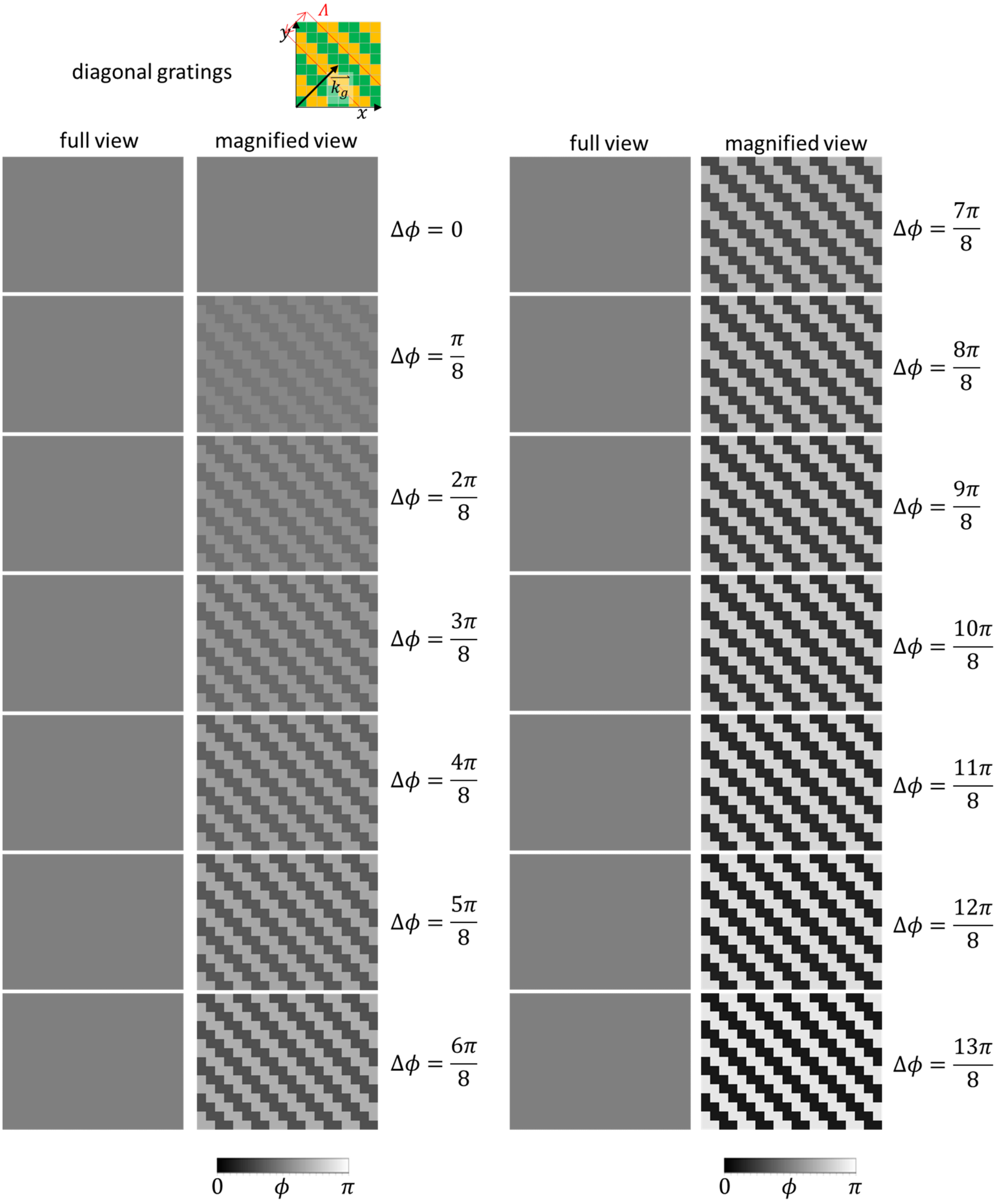

Figure S4. Diagonal phase gratings for the measurement of extract beam intensity as a function of phase depth $\Delta\phi$.

A phase grating is overlapped on the Gaussian beam, and the diffraction was filtered at the Fourier plane by a circular spatial frequency filter with $\Delta d = 4.0\text{mm}$. Fig.

S5 shows the beam profile observed with a different phase grating shown in Fig. S3 and S4. In terms of wrapped phase, 0-2π inherent to the SLM we used; a ring could be seen in the beam pattern. The beam intensity, which is integrated over a circle with a diameter of 1 mm inside of the ring, was plotted as a function of phase difference $\Delta\phi$, as shown in Fig. 7.

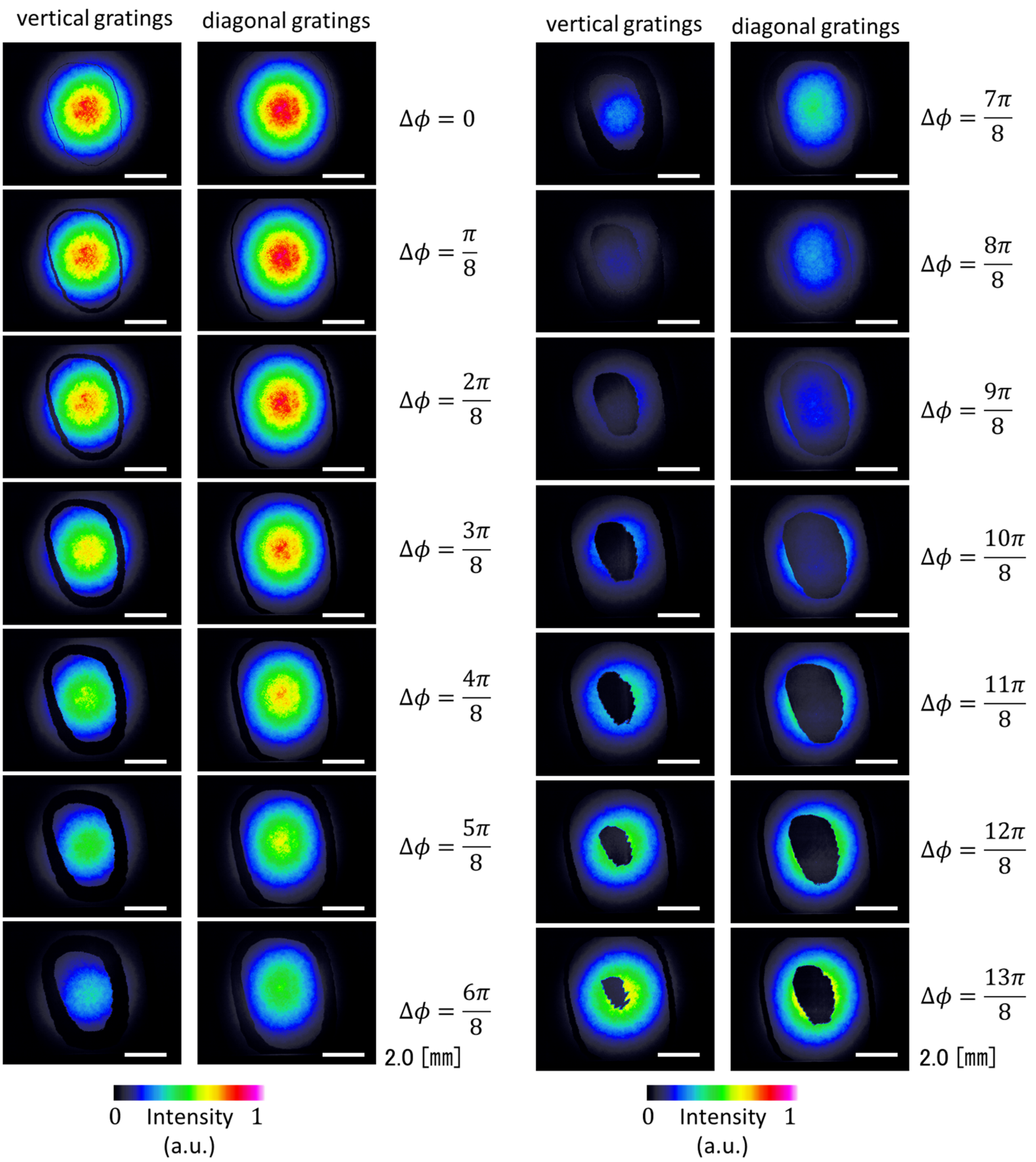

Figure S5. Extracted beam profiles with uniform vertical and diagonal phase gratings. White scale bars correspond to 2 mm.

The fitting curve was derived as follows. According to the theory, the electric field strength is linear to $\cos\left(\frac{\Delta\phi}{2}\right)$ as shown in Eq. (6). So, the following formula was fitted to the experimental data by least square fitting.

$$(\Delta\phi) = \mathrm{a}\ \cos^2(\mathrm{b}\ \ \Delta\phi + \mathrm{c}) + \mathrm{d} \qquad (7)$$

As a result, the curve formulas for vertical and diagonal gratings are obtained as follows;

$$I_{vertical}(\varDelta\phi) = 1.47 + 98.87\cos(3.16 - 0.38\varDelta\phi)^{2} \qquad (8)$$

$$I_{diagonal}(\varDelta\phi) = 3.16 + 97.11\cos(3.13 + 0.31\varDelta\phi)^{2} \qquad (9)$$

These curves are shown in Fig. S6. By using these fitting curves, phase grating for beam shaping was generated.

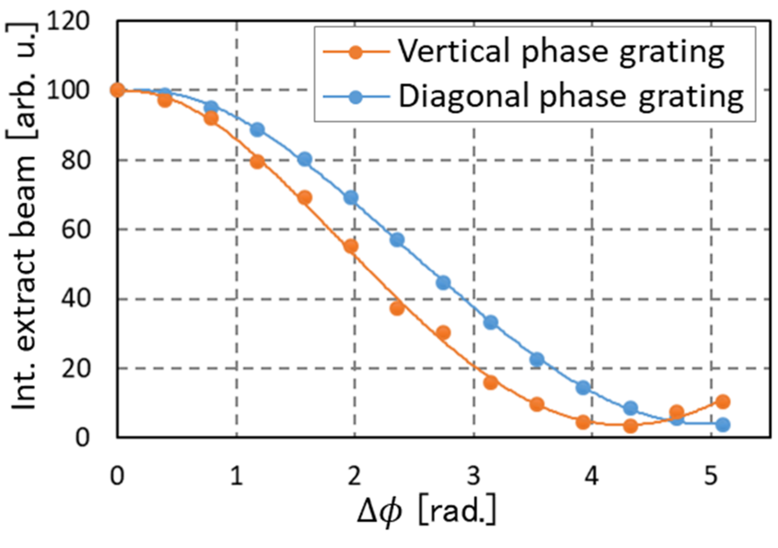

Figure S6. Intensity of the extracted beam as a function of the phase depth $\Delta\phi$.

The period of the vertical phase grating is $\varLambda = 80$ µm (4 pixels), and the period of the diagonal phase grating is $\varLambda = 56.6$ µm ($2\sqrt{2}$ pixels). The structures are shown

in Figs. 2a and 2b.

4. Numerical evaluation method for flatness and edge steepness

This calculation is compatible with ISO 13694:2000(E) Standard for laser beam measurements and the explanation follows the technical manual attached to the beam profiler[3]. The beam uniformity is defined by the normalized RMS (root mean square) deviation of the power density.

The beam uniformity range is zero to one with a perfectly flat top beam having a value of zero. To calculate this value, there are 3 steps:

I. Calculate the average power density of the portion of the beam exceeding the user-defined threshold.

II. Calculate the RMS deviation of the power density of the portion of the beam exceeding the user-defined threshold.

III. Calculate the ratio:

$$Beam\ \ Uniformity = \frac{RMS_{power\ \ density}}{AVG_{power\ \ density}} \qquad (10)$$

The user-defined threshold was set to 50% of the peak value.

Edge steepness is the normalized difference between two effective irradiation areas with intensity levels between 10% and 90% of the maximum intensity. The edge steepness range is zero to one with a flat top beam with vertical edges having a value of zero.

To find this value, there are 3 steps:

I. Find the area of the beam where the intensity is 10% (of the peak intensity) or greater. Call this A1.

II. Find the area of the beam where the intensity is 90% (of the peak intensity) or greater. Call this A2.

III. Normalize to A1 and find the difference as follows;

$$edge\ \ steepness = \frac{(A_1 - A_2)}{A_1} \tag{11}$$

Supplementary information accompanies the manuscript on the Light: Science & Applications website (http://www.nature.com/lsa/).

Supplementary References

1. Bagnoud, V. & Zuegel, J. D. Independent phase and amplitude control of a laser beam by use of a single-phase-only spatial light modulator. *Opt. Lett.* **29**, 295–297 (2004).
2. Osawa, K. *et al.* Beam shaping by spatial light modulator and 4 f system to square and top-flat for interference laser processing. *Proc. SPIE* **10091**, 100911C (2017).
3. Coherent. Technical manual of beam analyzer. (2018).
